# Dual‐Task Single‐Leg Hop Performance Exposes Persistent Asymmetry and Movement Deficits After Anterior Cruciate Ligament Reconstruction: A Randomised Crossover Experimental Study

**DOI:** 10.1155/tsm2/5079279

**Published:** 2026-07-15

**Authors:** Simon Valot, Ayrton Moiroux--Sahraoui, Adrien Cerrito, Florian Forelli

**Affiliations:** ^1^ Centre Sablais de L’Appareil Locomoteur, Les Sables D’Olonne, 85340, Pays de La Loire, France; ^2^ Orthopaedic Surgery Department, @OrthoLab, Clinic of Domont, Ramsay Healthcare, Domont, Ile de France, France; ^3^ Haute-Ecole Arc Santé, HES-SO University of Applied Sciences and Arts Western, Switzerland, Delémont, 2800, Switzerland

**Keywords:** anterior cruciate ligament reconstruction, cognitive–motor interaction, dual-task performance, limb symmetry index, movement quality, return-to-sport, single-leg hop

## Abstract

**Background:**

Dual‐task paradigms are increasingly used to investigate cognitive–motor interactions after anterior cruciate ligament reconstruction (ACLR), yet their effects on hop performance, movement quality and limb symmetry remain insufficiently quantified. The objective of this study was to compare these outcomes under single‐ and dual‐task conditions between individuals after ACLR and healthy controls.

**Methods:**

Thirty‐one participants (15 ACLR and 16 healthy controls) were included in this multicentre randomised repeated‐measures experimental study. Participants performed the single‐leg hop for distance (SLHD) under single‐task and dual‐task conditions using counterbalanced sequences. Movement quality was assessed using the Qualitative Assessment of Single‐Leg Landing Scale (QASLS). In the dual‐task condition, participants performed the hop while simultaneously completing a standardised visuo‐attentional working‐memory task. Dual‐task cost (DTC) was calculated for SLHD, QASLS and limb symmetry index (LSI). The primary outcome was SLHD DTC. Analyses were based on linear mixed‐effect models, with sequence‐specific findings reported descriptively to support transparency regarding the counterbalanced design.

**Results:**

Under dual‐task conditions, SLHD DTC was numerically higher in the ACLR group than in controls in sequences A1 (6.5 ± 6.5% vs 2.7 ± 6.9%; *p* = 0.227) and A2 (1.1 ± 1.1% vs −0.7 ± 6.8%; *p* = 0.316), although these between‐group differences were not statistically significant. QASLS DTC was not significantly greater in the ACLR group in sequence A1 (0.19 ± 0.23 vs 0.12 ± 0.36 points; *p* = 0.385) or A2 (0.43 ± 0.28 vs 0.56 ± 0.87 points; *p* = 0.613). The largest between‐group differences were observed for LSI DTC, which was significantly higher in the ACLR group in sequences A1 (3.8 ± 1.9% vs 0.7 ± 1.3%; *p* < 0.001) and A2 (6.5 ± 2.4% vs 0.6 ± 1.2%; *p* < 0.001). Repeated‐condition sequences B1 and B2 were retained to explore potential order, learning and fatigue effects and were not interpreted as DTC sequences. Sequence‐specific descriptive findings in the ACLR group showed dual‐task‐related effects for limb symmetry in A1 and A2, whereas controls showed minimal changes.

**Conclusion:**

Dual‐task conditions were associated with measurable changes in SLHD performance, movement quality and limb symmetry after ACLR.

**Trail Registration:** Clinical trial.gov.identifier: NCT06768957

## 1. Introduction

Anterior cruciate ligament reconstruction (ACLR) aims to restore joint stability and allow a safe return to sport after rupture, yet persistent neuromuscular deficits often remain despite rehabilitation programmes that meet conventional clinical benchmarks (e.g. strength recovery, limb symmetry indices [LSIs] and return‐to‐sport [RTS] clearance), which may not fully capture neurocognitive and sensorimotor recovery [1, 2]. Conventional RTS assessments typically focus on muscle strength, hop distance and LSIs, assuming that symmetrical performance equates to full recovery [3–5]. However, these metrics primarily capture mechanical outputs and overlook the neurocognitive aspects of motor control that are essential for reactive and unpredictable sporting environments [6–8].

Growing evidence suggests that ACLR disrupts not only peripheral function but also cortical organisation, affecting proprioceptive processing, attentional control and sensorimotor integration [9, 10]. Functional neuroimaging and electroencephalography studies reveal increased activation of prefrontal and sensorimotor regions during motor tasks in individuals with ACLR, indicating a compensatory shift from automatic to consciously controlled motor strategies [6, 7, 9]. This reliance on higher cognitive resources implies that attentional load may directly influence performance, particularly during complex or dual‐task situations where attention must be divided [11, 12].

Dual‐task paradigms, combining a primary motor task with secondary cognitive demand, provide a useful framework for evaluating the interaction between cognition and motor control [13, 14]. Such approaches have been applied in balance control, gait and reactive agility to simulate the attentional demands of sport [3, 5, 15–17]. In healthy athletes, dual‐task interference tends to be minimal, reflecting efficient automaticity and flexible allocation of attentional resources [13, 16, 18]. Conversely, in individuals recovering from ACLR, adding a cognitive load may magnify latent deficits in coordination and control [19–21]. These findings underline the potential of dual‐task assessment to reveal neuromotor limitations that remain hidden during single‐task testing.

The single‐leg hop for distance (SLHD) is a widely used clinical measure of lower‐limb function after ACLR due to its simplicity, reproducibility and functional relevance. While it captures essential components of sport‐related movements, such as unilateral propulsion and landing control, it remains a highly controlled and predictable task, lacking the perceptual–cognitive and reactive demands inherent to real‐world sport situations. [4, 22, 23]. However, traditional SLHD protocols are conducted under single‐task conditions, which may not replicate the cognitive–motor complexity of sport. Introducing a concurrent cognitive task could enhance the ecological validity of hop testing by approximating real‐world demands, where athletes must simultaneously process environmental information and execute rapid, coordinated movements [6, 24]. Furthermore, qualitative assessment tools, such as the Qualitative Assessment of Single‐Leg Landing Scale (QASLS), allow clinicians to quantify movement quality and frontal‐plane control—factors directly linked to injury risk and neuromuscular competence [25, 26].

Despite its relevance, the dual‐task framework remains underexplored in late‐stage ACLR rehabilitation. Most studies have investigated balance or gait performance, whereas few have examined power‐based functional tests or combined these with validated qualitative scoring [3, 5, 11]. Moreover, evidence on whether cognitive interference amplifies interlimb asymmetry or alters movement patterns during unilateral hopping is limited. Addressing this gap could improve clinicians’ ability to detect residual deficits that increase reinjury risk and refine RTS decision‐making criteria [1, 8, 27].

The six‐month postoperative period represents a clinically relevant transition point in ACLR rehabilitation. At this stage, many athletes begin or prepare for progressive sport‐specific participation, while residual deficits in strength, limb symmetry, neuromuscular control and cognitive–motor integration may still be present [28–30]. Assessing dual‐task hop performance at this time point may therefore help identify deficits that are not captured by conventional single‐task RTS assessments.

Therefore, the present multicentre, randomised comparative study aimed to evaluate the effect of a concurrent cognitive load on SLHD performance, movement quality and limb symmetry in individuals 6 months after ACLR compared with healthy controls. It was hypothesised that (1) adding a cognitive task would reduce hop distance and movement quality scores in both groups, and (2) these effects would be more pronounced in the ACLR group due to persistent neurocognitive and neuromuscular deficits. By exploring the dual‐task cost (DTC) on quantitative and qualitative metrics, this study sought to contribute new evidence supporting the integration of cognitive–motor assessment in late‐phase rehabilitation and RTS evaluation after ACLR.

## 2. Methods

### 2.1. Study Design and Ethical Approval

This multicentre randomised crossover repeated‐measures experimental study was designed to evaluate the influence of a concurrent cognitive load on SLHD performance after ACLR. The study was classified as *Recherche Impliquant la Personne Humaine—catégorie 3 (RIPH-3)* and conducted according to the Declaration of Helsinki and adapted from the STrengthening the Reporting of OBservational studies in Epidemiology (STROBE).

The protocol was approved by the Comité de Protection des Personnes Île‐de‐France III (CPP No. SI 24.04567.000755, approval date 17 December 2024), authorised by the Agence Nationale de Sécurité du Médicament et des Produits de Santé (ANSM No. 2024‐A02038‐39), and declared to the French data‐protection authority (CNIL, methodological reference MR‐003). All participants provided written informed consent before inclusion [31, 32].

### 2.2. Participants

**FIGURE 1:**
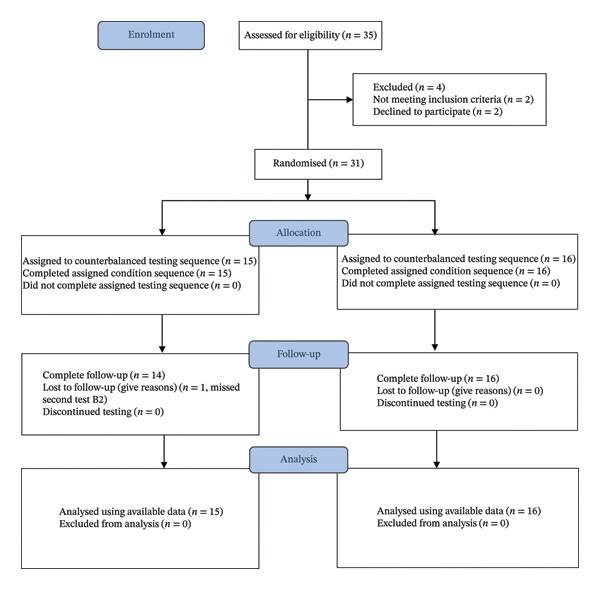
Participant flow diagram.

Two groups were recruited between January and June 2025 (Figure 1). The study was conducted in France across three outpatient rehabilitation centres involved in ACLR rehabilitation and one university‐based recruitment site for healthy controls. ACLR participants were recruited and assessed at Cabinet Madini, Poitiers, France; Centre Sablais de l’Appareil Locomoteur, Les Sables d’Olonne, France; and Centre Orthosport, Domont, France. Healthy control participants were recruited from the University of Poitiers, Poitiers, France, and assessed using the same experimental procedures. All testing was performed indoors on a nonslip sport surface using standardised instructions and measurement procedures.

The ACLR group consisted of individuals who had undergone primary unilateral ACLR using an autograft technique. Inclusion criteria were a unilateral, noncontact ACL rupture treated with an autograft of hamstring or bone–patellar tendon–bone; a postoperative interval of 6 ± 1 months; age between 16 and 40 years; and a Tegner activity score ≥ 5. Participants were excluded if they had concomitant ligament injuries requiring surgical management, a revision ACLR, previous significant lower‐limb injury or surgery other than the index ACLR, a contact mechanism of injury, lower‐limb injury within the previous 12 months unrelated to the index ACL injury, a Tegner score < 5 or time since ACLR outside the inclusion window. Those who declined participation, withdrew consent or were lost to follow‐up were also excluded. The control group consisted of healthy participants matched for age, sex distribution and physical activity level (Tegner ≥ 5) without any history of lower‐limb trauma during the preceding 6 months. Control participants were excluded if they reported previous lower‐limb surgery, significant lower‐limb injury within the previous 12 months, current pain or injury affecting hop performance, neurological disorders or vestibular disorders.

Baseline data recorded for both groups included age, sex, height, body mass, limb dominance, side of reconstruction, time since surgery, graft type and main sporting activity. Limb dominance was defined as the preferred leg for kicking and is referred to as the dominant limb (DL) and non‐dominant limb (NDL). In the ACLR group, the operated limb (OL) and non‐operated limb (NOL) were identified separately to allow within‐participant comparisons [33, 34].

Rehabilitation history was collected for ACLR participants when available, including time since surgery, graft type, rehabilitation centre, approximate rehabilitation duration, weekly treatment frequency, and exposure to strengthening, neuromuscular control, plyometric, running and sport‐specific exercises [15, 35–38]. Rehabilitation was delivered according to local clinical practice rather than a fully standardised research protocol [28, 29]. Across centres, all participants completed conventional postoperative rehabilitation including range‐of‐motion recovery, progressive strengthening, balance and neuromuscular exercises and gradual functional progression. However, the exact content, intensity and progression criteria varied between centres and were therefore considered a potential source of clinical heterogeneity.

### 2.3. Sample Size Calculation

An a priori sample size calculation was performed for the primary outcome, SLHD DTC (%), defined as the relative percentage reduction in single‐leg hop distance from the single‐task to the dual‐task condition. At the time of study planning, no robust preliminary data were available for this exact SLHD DTC outcome in individuals after ACLR compared with healthy controls. Therefore, the calculation was based on a conservative large between‐group effect size assumption (Cohen’s *d* = 0.80), informed by previous dual‐task and neurocognitive functional testing studies in ACLR populations reporting moderate‐to‐large cognitive–motor interference effects, while avoiding reliance on more optimistic estimates from pilot or nonidentical paradigms.

Using a two‐sided alpha level of 0.05% and 80% statistical power, approximately 26 participants per group were required to detect a between‐group difference in SLHD DTC. Due to recruitment feasibility constraints, strict eligibility criteria and the narrow postoperative inclusion window, the final sample included 15 ACLR participants and 16 healthy controls. The study should therefore be interpreted as exploratory and hypothesis‐generating, particularly for secondary outcomes and interaction effects.

### 2.4. Randomisation

The study used a mixed factorial design combining a between‐subject factor (Group: ACLR vs control) and a within‐subject factor (Condition: single‐task vs dual‐task). Each participant performed both conditions within a single session.

The order of testing conditions (single‐task and dual‐task) was randomised via four counterbalanced sequences (A1, A2, B1 and B2). Sequences A included two different conditions between session 1 and session 2 (single‐task block and dual‐task block), whereas sequences B included the same sequence from one session to the next.

Sequences A and B were implemented as part of the counterbalanced experimental design to control for potential order, learning and fatigue effects associated with repeated testing under single‐task and dual‐task conditions. These sequences did not represent separate experimental groups but rather different testing orders randomly assigned to participants. The inclusion of B sequences therefore aimed to preserve internal validity by minimising systematic bias related to task exposure order.

Sequence assignment was computer‐generated in permuted blocks of four, stratified by centre and concealed using sequentially numbered, opaque, sealed envelopes prepared by an independent researcher. Because the motor–cognitive manipulation was apparent, participants and on‐site assessors were not blinded to the condition performed; however, video‐based QASLS scoring was conducted by independent raters blinded to group, condition and limb, and all data processing and statistical analyses used anonymised identifiers to minimise bias [32].

Analyses used an all‐available‐data approach. One ACLR participant missed the second session in sequence B2; available single‐task data were retained for raw outcome analyses, but no DTC was calculated for B2 because this sequence involved repeated single‐task assessments.

### 2.5. Procedure

**FIGURE 2:**
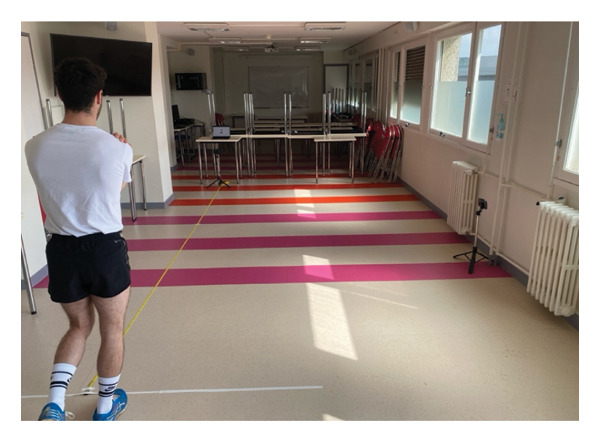
Spatial organisation of the SLHD.

All assessments were conducted indoors on a nonslip sport surface under stable ambient conditions. Participants wore standard indoor footwear and sport attire. Before testing, each participant completed a standardised warm‐up of 5 minutes of low‐intensity cycling, dynamic lower‐limb mobility and three submaximal familiarisation SLHD per limb. Standardised instructions were read verbatim by the assessor: participants were to perform a maximal SLHD with hands placed on the shoulders to minimise arm swing and standardise upper‐body contribution, to stabilise the landing without additional hops or balance‐recovery steps and to report any pain precluding maximal effort (Figure 2). Distance was measured from the toe at take‐off to the heel at first ground contact with a calibrated tape aligned to the hop vector, consistent with established SLHD procedures and reliability reports [22, 23, 39]. Trials were declared invalid if balance was lost, if an extra hop occurred, if the prescribed hand position was violated or if pain incompatible with maximal effort was reported; invalid trials were repeated after the same intertrial rest.

A 1‐week interval between the two sessions was established to assess the two experimental conditions. In the single‐task condition, participants performed the SLHD alone. In the dual‐task condition, participants performed the same SLHD while simultaneously completing a standardised visuo‐attentional working‐memory task. Immediately before the hop command, a five‐digit sequence was visually presented to the participant on a printed card or digital screen positioned at eye level. The sequence was displayed for 3 s, after which the participant initiated the hop following the assessor’s verbal cue. Participants were instructed to memorise the digit sequence and reproduce it in reverse order during or immediately after landing stabilisation. Cognitive performance was scored as the number of digits correctly recalled in the correct reverse order. Cognitive errors were recorded for each valid trial and were defined as incorrect digit recall, incorrect order, omission or failure to provide a response.

The same stimulus length, presentation timing, verbal instructions and scoring rules were used across centres. This task was selected because backward digit recall imposes working‐memory, visual‐attentional and executive‐control demands that are relevant to cognitive–motor interference during RTS situations after ACLR. Although this exact SLHD dual‐task protocol has not been specifically validated in ACLR populations, it was adapted from previously published dual‐task paradigms used to investigate cognitive–motor interference during functional lower‐limb task [40–43].

Video recordings were simultaneously obtained in frontal and sagittal planes using digital cameras mounted on 60‐cm tripods positioned at fixed distances from the take‐off line to preserve the field of view for subsequent qualitative scoring [25, 26].

In the ACLR group, limb designation followed surgical status so that the OL and the NOL were explicitly contrasted within participants. Participants began the test with the NOL in order to get themselves with the hop and then continued with the OL. Each participant followed one of four prerandomised execution orders designed to minimise order, learning, fatigue and cognitive interference effects. Sequences A1 and A2 were crossover sequences in which both experimental conditions were performed within the same participant. In sequence A1, the dual‐task condition was performed first, followed by the single‐task condition, whereas sequence A2 began with the single‐task condition followed by the dual‐task condition. In contrast, sequences B1 and B2 were repeated‐condition sequences: B1 included two dual‐task assessments, whereas B2 included two single‐task assessments. These B sequences were retained to explore potential order, learning and fatigue effects, but were not interpreted as DTC sequences. Within each block, participants attempted up to three valid SLHD with 45 s of rest between trials to limit fatigue while preserving a consistent neuromotor state; the best (longest) distance was retained for that limb‐condition pairing, and movement quality errors and cognitive‐error counts (for dual‐task) were recorded in parallel [23, 39].

The control group followed the identical sequencing logic. Limb designation mapped to limb dominance: accordingly, participants began the test with the DL in order to get themselves with the hop and then continued with the NDL. This mapping ensured that every participant in both cohorts completed both conditions on both limbs in a counterbalanced, two‐session design, while preserving clinical relevance to the reconstructed knee in ACLR and to functional dominance in controls.

Movement quality at take‐off and landing was assessed on the recorded videos using the QASLS for unilateral landing tasks, applying published scoring procedures and reliability guidance [26, 44]. For each limb–condition pairing, the primary quantitative metric was the best distance (metres) across the three valid trials. The LSI % was computed within condition as 100 × (OL ÷ NOL) in the ACLR group and 100 × (NDL ÷ DL) in the control group [5, 30].

Raw SLHD distance, QASLS score and LSI were recorded for each valid limb–condition pairing. DTC was subsequently derived only for crossover sequences in which both single‐task and dual‐task conditions were available within the same participant.

### 2.6. Outcomes

The primary outcome was the DTC for single‐leg hop distance (SLHD DTC, %), defined as the relative percentage reduction in hop distance from the single‐task to the dual‐task condition. Secondary outcomes included QASLS DTC, LSI DTC, raw SLHD distance, QASLS score and LSI values under single‐task and dual‐task conditions.

### 2.7. Statistical Analysis

All statistical analyses were performed using Jamovi (v.2.5) and SPSS (v.29, IBM Corp., Armonk, NY, USA). The overall significance level was set at *α* = 0.05 (two‐tailed).

Model assumptions were assessed using residual plots, Q–Q plots of model residuals and inspection of influential observations. Linear mixed‐effects models were used as the primary analytical approach to account for the repeated‐measures and crossover structure of the study. Separate models were fitted for SLHD distance, QASLS score and LSI. For each model, Group (ACLR vs Control), Condition (single‐task vs dual‐task), Sequence/order and Centre were entered as fixed effects. The Group × Condition interaction was used to determine whether the effect of dual‐task loading differed between ACLR participants and healthy controls. Participant was included as a random intercept to account for within‐participant correlation across repeated measurements.

For DTC outcomes, separate models were fitted for SLHD DTC, QASLS DTC and LSI DTC. These models included Group, Sequence/order and Centre as fixed effects. Participant was included as a random intercept when repeated DTC observations were available. Estimated marginal means, mean differences, 95% confidence intervals (CIs) and *p* values were reported for the main model‐based comparisons. For descriptive sequence‐specific comparisons, effect sizes were reported as Cohen’s *d*.

Sequence/order was included in the models as an adjustment factor to account for the counterbalanced design. Sequences were not considered separate experimental groups, and sequence‐specific findings were interpreted descriptively.

When model assumptions were not adequately met, sensitivity analyses using nonparametric or robust approaches were performed. Sequence‐specific descriptive results were retained to support transparency regarding the counterbalanced design, but the main interpretation was based on the mixed‐effects models.

The analysis plan followed a mixed factorial structure, combining a within‐subject factor (*Condition*: single‐task vs dual‐task) and a between‐subject factor (*Group*: ACLR vs Control). In addition, Sequence (A vs B, corresponding respectively to sessions evaluating the two different conditions or the same condition twice, between single‐ or dual‐task conditions) and Centres were entered as fixed factors to account for order and site‐related effects.

For each main outcome—SLHD, QASLS score and LSI—results are reported as mean ± standard deviation (SD), together with mean differences (Δ), 95% CI, *p* values and standardised effect sizes where applicable. For descriptive sequence‐specific comparisons, effect sizes are reported as Cohen’s *d*. The interpretation of *d* followed conventional thresholds: trivial (< 0.20), small (0.20–0.49), moderate (0.50–0.79) and large (≥ 0.80) [6, 7].

Given the study’s sequential design, descriptive results were additionally presented per sequence (A1–B2) to allow transparency with the randomisation structure. Sequence‐specific results were therefore considered descriptive or sensitivity analyses and were not used as the primary basis for statistical interpretation.

To address the impact of the cognitive interference, DTC was calculated only for the crossover sequences in which both single‐task and dual‐task measurements were available within the same participant, namely, A1 and A2. For SLHD distance and LSI, DTC was expressed in percentage as

(1) DTC%=100×Dual−SingleSingle.

For QASLS, DTC was expressed in points (Dual − Single). LSI was computed within each condition as 100 × (OL ÷ NOL) in the ACLR group and 100 × (NDL ÷ DL) in the Control group before deriving DTC. DTC values were summarised by mean ± SD for each sequence. Between‐group differences in DTC outcomes were analysed using the mixed‐effects models described above. Sequences B1 and B2 were not used to calculate DTC because they involved repeated assessments of the same condition.

Missing data were handled according to an all‐available‐data approach. One ACLR participant in sequence B2 did not attend the second testing session. Available single‐task data were retained for analyses of raw outcomes, but no DTC was calculated for B2 because this sequence involved repeated single‐task assessments.

For confirmatory contrasts (prespecified primary outcomes and their Group × Condition interaction), Holm’s correction was applied to control the familywise error rate. All other analyses were considered exploratory and are presented with unadjusted *p* values for transparency.

Effect sizes were reported as Cohen’s *d* for descriptive sequence‐specific comparisons. Mean differences and 95% CIs were reported for all main comparisons. For between‐group comparisons, mean differences were calculated as ACLR minus Control. For condition effects, mean differences were calculated as dual‐task minus single‐task. For DTC outcomes, mean differences were calculated as ACLR minus Control. Sequence‐specific results were interpreted descriptively, whereas the main statistical interpretation was based on the mixed‐effects models.

Statistical results were interpreted jointly based on *p* values, 95% CIs and effect sizes to balance statistical significance, practical relevance and precision of estimation.

## 3. Results

A total of 31 participants (Table 1) were included in the analysis: 15 patients in the ACLR group (test group) and 16 healthy participants in the control group.

**TABLE 1:** Demographic data.

Variable	ACLR group (*n* = 15) Mean ± SD	Control group (*n* = 16) Mean ± SD	*p*‐value
Age (years)	25.8 ± 4.7	24.9 ± 3.9	0.54
Sex (M/F)	10/5	11/5	—
Height (cm)	176.2 ± 7.9	175.4 ± 8.1	0.73
Body mass (kg)	72.3 ± 9.4	70.8 ± 10.2	0.61
BMI (kg/m^2^)	23.3 ± 2.1	22.9 ± 1.8	0.67
Dominant leg (right/left)	13/2	14/2	—
Operated leg (right/left)	8/7	—	—
Postoperative time (months)	6.2 ± 0.5	—	—
Activity level (Tegner scale)	6.1 ± 1.2	6.3 ± 1.0	0.59

Abbreviations: BMI = body mass index, SD = standard deviation.

All participants met the inclusion criteria and completed the experimental procedures. One participant in the ACLR group (sequence B2) did not attend the second testing session; however, the first SLHD dataset was retained for analysis according to the all‐available‐data approach.

Participants in the ACLR group were tested at a mean of 6.1 ± 0.8 months after ACLR. All reconstructions were primary, unilateral and isolated ACLR using autograft techniques (hamstring and bone patellar tendon graft). The two groups were comparable for age, sex and activity level (Tegner ≥ 5).

### 3.1. ACLR Group Descriptive Analysis

**FIGURE 3:**
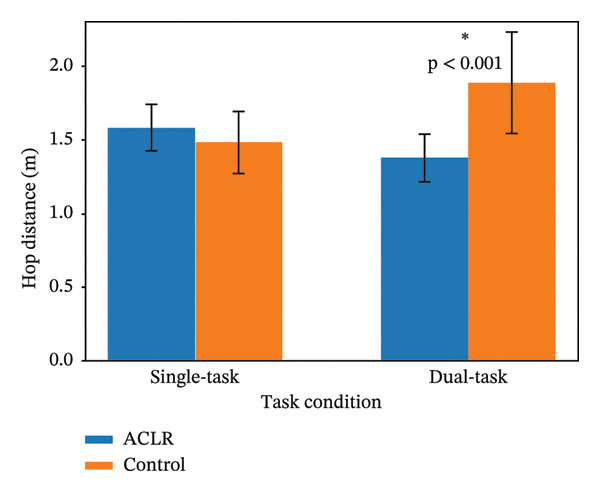
Mean single‐leg hop distance (SLHD) values under single‐task (ST) and dual‐task (DT) conditions across experimental sequences in the ACLR and control groups. Bars represent mean ± standard deviation. ^∗^ indicates a significant between‐group difference (*p* < 0.05).

Within the ACLR group (Figure 3), comparisons between the single‐task and dual‐task conditions revealed limited changes in SLHD performance across sequences. A significant reduction was observed only in sequence B2, where SLHD decreased from 1.376 ± 0.161 m to 1.351 ± 0.209 m (Δ = −0.025 m; *p* = 0.024).

For movement quality (QASLS), a significant alteration was identified in sequence A2, with scores increasing from 4.000 ± 1.871 to 5.536 ± 1.347 points (Δ = +1.536 points; *p* = 0.006), whereas no meaningful differences were observed in the other sequences.

LSI values were significantly reduced under dual‐task conditions in sequence A1 (Δ = −3.8%; *p* = 0.031), sequence A2 (Δ = −6.5%; *p* = 0.016) and sequence B2 (Δ = −8.1%; *p* = 0.004). No significant change was observed in sequence B1. Detailed descriptive statistics and effect sizes are presented in Table 2.

**TABLE 2:** Sequence‐specific descriptive between‐group comparisons of SLHD, QASLS and LSI across counterbalanced testing sequences (A1–B2).

Outcome	Sequence	Condition	ACLR group (mean ± SD)	Control group (mean ± SD)	Δ	95% CI (lower; upper)	*p* value	Cohen’s d
SLHD (m)	A1	DT	1.582 ± 0.158	1.482 ± 0.209	+0.100	(−0.036; 0.236)	0.143	0.54
		ST	1.692 ± 0.190	1.545 ± 0.215	+0.147	(−0.004; 0.298)	0.056	0.72
	A2	DT	1.701 ± 0.148	1.505 ± 0.369	+0.196	(−0.010; 0.402)	0.061	0.68
		ST	1.714 ± 0.175	1.459 ± 0.375	+0.255	(0.041; 0.469)	0.021	0.86
	B1	DT	1.557 ± 0.354	1.693 ± 0.380	−0.136	(−0.410; 0.138)	0.322	−0.37
		ST	1.526 ± 0.378	1.690 ± 0.378	−0.164	(−0.442; 0.114)	0.237	−0.43
	B2	DT	1.376 ± 0.161	1.887 ± 0.343	−0.511	(−0.709; −0.313)	< 0.001	−1.89
		ST	1.351 ± 0.209	1.890 ± 0.360	−0.539	(−0.753; −0.325)	< 0.001	−1.85

QASLS (points)	A1	DT	5.107 ± 1.286	3.923 ± 2.018	+1.184	(−0.058; 2.426)	0.061	0.69
		ST	4.500 ± 1.253	3.655 ± 1.717	+0.845	(−0.250; 1.940)	0.124	0.56
	A2	DT	5.536 ± 1.347	4.615 ± 1.878	+0.921	(−0.301; 2.143)	0.134	0.56
		ST	4.000 ± 1.871	3.731 ± 2.089	+0.269	(−1.186; 1.724)	0.708	0.14
	B1	DT	5.077 ± 1.765	4.741 ± 2.068	+0.336	(−1.084; 1.756)	0.631	0.18
		ST	4.741 ± 2.068	5.077 ± 1.765	−0.336	(−1.756; 1.084)	0.631	−0.18
	B2	DT	4.481 ± 1.929	4.704 ± 1.589	−0.223	(−1.530; 1.084)	0.729	−0.13
		ST	4.704 ± 1.589	4.481 ± 1.929	+0.223	(−1.084; 1.530)	0.729	0.13

LSI (%)	A1	DT	91.7 ± 7.1	97.8 ± 4.1	−6.1	(−10.45; −1.75)	0.008	−1.06
		ST	87.9 ± 8.5	97.1 ± 4.5	−9.2	(−14.32; −4.08)	< 0.001	−1.35
	A2	DT	92.9 ± 6.3	96.5 ± 5.2	−3.6	(−7.87; 0.67)	0.095	−0.63
		ST	86.4 ± 7.9	95.9 ± 5.0	−9.5	(−14.47; −4.53)	< 0.001	−1.43
	B1	DT	88.6 ± 9.4	97.5 ± 4.2	−8.9	(−14.26; −3.54)	0.002	−1.22
		ST	90.3 ± 8.0	98.2 ± 3.9	−7.9	(−12.66; −3.14)	0.002	−1.27
	B2	DT	93.8 ± 6.5	97.9 ± 4.6	−4.1	(−8.29; 0.09)	0.055	−0.73
		ST	85.7 ± 8.1	97.3 ± 4.8	−11.6	(−16.73; −6.47)	< 0.001	−1.73

*Note: Δ*; mean difference calculated as ACLR − Control; values are presented as mean ± SD. Mean differences were calculated as ACLR minus Control. Sequence‐specific results are presented descriptively to support transparency regarding the counterbalanced design. A1 and A2 were crossover sequences including both single‐task and dual‐task conditions and were used to calculate dual‐task cost. B1 and B2 were repeated‐condition sequences used to explore potential order, learning and fatigue effects and were not interpreted as dual‐task cost sequences. The main statistical interpretation was based on the mixed‐effects models.

Abbreviations: ACLR = anterior cruciate ligament reconstruction, CI = confidence interval, LSI = limb symmetry index, QASLS = Qualitative Analysis of Single‐Leg Loading, SD = standard deviation, SLHD = single‐leg hop for distance.

### 3.2. Control Group Descriptive Analysis

Within the control group (Figure 3), descriptive sequence‐specific findings showed no significant differences between single‐task and dual‐task conditions in SLHD performance or LSI values across sequences (Table 2), while the main statistical interpretation was based on the mixed‐effects models.

For movement quality (QASLS), a significant difference was observed only in sequence B2, where scores decreased from 4.704 ± 1.589 to 4.481 ± 1.929 points under dual‐task conditions (Δ = −0.223 points; *p* = 0.004). No other significant changes were identified across the remaining sequences.

Detailed descriptive statistics and effect sizes are presented in Table 2.

### 3.3. Intergroup Analysis

Across sequences (Table 2), between‐group comparisons for SLHD revealed a significant reduction in hop distance for the ACLR group only in sequence B2, where values were 1.376 ± 0.161 m in the ACLR group versus 1.887 ± 0.343 m in controls (Δ = −0.511 m; *p* < 0.001, *d* = −1.89). No other significant between‐group differences were observed for SLHD.

For movement quality (QASLS), no significant between‐group differences were identified across sequences, although a trend towards higher QASLS scores in the ACLR group was observed in sequence A1 (Δ = +1.184 points; *p* = 0.061, *d* = 0.69).

For LSI, significantly lower values were observed in the ACLR group in sequence A1 (91.7 ± 7.1% vs 97.8 ± 4.1%; Δ = −6.1%; *p* = 0.008, *d* = −1.06) and sequence B1 (90.3 ± 8.0% vs 98.2 ± 3.9%; Δ = −7.9%; *p* = 0.002, *d* = −1.27). No significant between‐group differences were observed in sequences A2 or B2.

Detailed descriptive statistics, CIs and effect sizes are presented in Table 2.

### 3.4. DTC

DTC analyses were restricted to the crossover sequences A1 and A2, in which both single‐task and dual‐task conditions were available within the same participant. Sequences B1 and B2 were repeated‐condition sequences and were therefore not interpreted as DTC sequences.

For SLHD distance, DTC values were numerically higher in the ACLR group than in controls in A1 (6.5 ± 6.5% vs 2.7 ± 6.9%) and A2 (1.1 ± 1.1% vs −0.7 ± 6.8%). However, between‐group differences were not statistically significant in either A1 (*p* = 0.227) or A2 (*p* = 0.316).

For QASLS, DTC values were small and did not differ significantly between groups. In A1, QASLS DTC was 0.19 ± 0.23 points in the ACLR group and 0.12 ± 0.36 points in controls (*p* = 0.385). In A2, QASLS DTC was 0.43 ± 0.28 points in the ACLR group and 0.56 ± 0.87 points in controls (*p* = 0.613).

**FIGURE 4:**
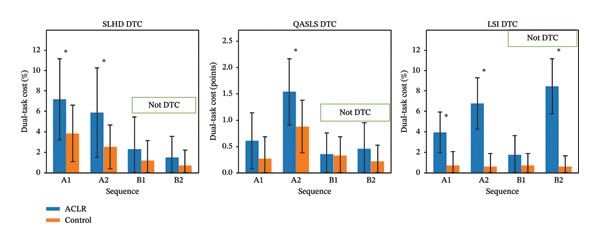
Mean dual‐task cost (DTC) values for single‐leg hop distance (SLHD), Qualitative Assessment of Single‐Leg Landing Scale (QASLS) and limb symmetry index (LSI) in crossover sequences A1 and A2. Bars represent mean ± standard deviation. ^∗^ indicates a significant between‐group difference (*p* < 0.05). Repeated‐condition sequences B1 and B2 were retained to explore potential order, learning and fatigue effects but were not interpreted as DTC sequences.

The largest between‐group differences were observed for LSI DTC. Significant increases were found in the ACLR group for sequence A1 (3.8 ± 1.9% vs 0.7 ± 1.3%; *p* < 0.001) and sequence A2 (6.5 ± 2.4% vs 0.6 ± 1.2%; *p* < 0.001). Sequences B1 and B2 were repeated‐condition sequences and were therefore not interpreted as DTC sequences. Detailed descriptive statistics and effect sizes are presented in Figure 4.

## 4. Discussion

This study examined how adding a cognitive load influences single‐leg hop performance, interlimb symmetry and movement quality in individuals 6 months after ACLR, compared with healthy controls. The six‐month postoperative assessment was intentionally selected because it represents a clinically relevant transition period in ACLR rehabilitation. At this stage, many athletes begin or prepare for progressive sport‐specific participation, while residual deficits in limb symmetry, neuromuscular control and cognitive–motor integration may still be present. These findings should therefore not be interpreted as indicating readiness or nonreadiness for unrestricted return to sport at 6 months. Rather, they suggest that this transition period may reveal persistent cognitive–motor deficits when functional tasks are performed under divided attention. Using a counterbalanced mixed design, all participants performed both single‐task and dual‐task conditions, enabling an integrated analysis of motor–cognitive interference. The main findings indicate that cognitive loading was associated with sequence‐specific changes in functional performance and movement quality, with the most consistent between‐group differences observed for limb symmetry under dual‐task conditions.

The reduction in hop distance under dual‐task conditions among ACLR participants highlights the persistence of neuromuscular control deficits during late rehabilitation. This decline is consistent with evidence that ACL‐injured individuals exhibit heightened dependence on cortical resources for movement control [6, 7]. Functional magnetic resonance imagery and electroencephalograms studies show increased frontal and sensorimotor activation patterns during simple motor tasks after ACLR, suggesting a shift from automatic to consciously controlled movement strategies [9, 10, 45]. Consequently, the addition of a secondary cognitive task may compete for limited attentional resources, disrupting movement planning and coordination. These findings are in line with experimental dual‐task paradigms demonstrating reductions of 5%–12% in vertical or horizontal jump distance in ACLR populations under cognitive interference [3].

Movement quality, as assessed by the QASLS, also deteriorated under dual‐task conditions, particularly in sequences A2 and B2, where participants displayed altered frontal‐plane alignment and trunk control. Previous kinematic investigations have shown similar degradation in lower‐limb mechanics under cognitive distraction, with increased dynamic valgus, reduced knee flexion angles and altered trunk motion [46, 47]. Electromyography‐based analyses confirm that concurrent cognitive demands reduce hamstring activation and co‐contraction ratios, weakening dynamic joint stability [13, 48, 49]. The QASLS, which has demonstrated good‐to‐excellent reliability [25, 26], therefore provides a clinically accessible means of quantifying the impact of dual‐task interference on movement coordination without laboratory instrumentation.

The decline in LSI under cognitive load reinforces the concept of ‘latent asymmetry’—deficits that remain undetected under simple conditions but become apparent when task demands increase [5, 30, 50]. While participants may present symmetrical hop distances in single‐task testing, their asymmetry reappears when attentional focus is divided, reflecting incomplete sensorimotor reintegration between limbs. This pattern supports previous work demonstrating that even after strength and performance metrics normalise, interlimb differences persist in cortical excitability, proprioceptive acuity and neuromuscular timing [27, 51–54]. Such findings underscore that restoration of automaticity, not merely of maximal performance, is a key objective of late‐stage rehabilitation.

The control group’s stable performance across conditions suggests that the dual‐task decrement observed in the ACLR group was not due to task novelty or measurement artefacts but reflects genuine neurocognitive constraints. Healthy athletes demonstrate more efficient neural resource allocation and reduced attentional cost during automated movements [16, 55]. Their ability to maintain symmetrical and coordinated patterns under cognitive load highlights the adaptive benefit of intact sensorimotor integration and attentional flexibility.

Collectively, these findings support the growing view that motor performance after ACLR cannot be fully understood without considering cognitive–motor interactions [6, 24]. Dual‐task paradigms provide an ecologically valid approach to evaluate the readiness for sport‐specific environments, where athletes must make rapid perceptual and decision‐making processes under physical stress. These results indicate that at 6 months postsurgery, ACLR participants still show measurable performance decrements under divided attention, suggesting that rehabilitation at this stage should not only target strength and symmetry but also incorporate neurocognitive and dual‐task training elements [11, 30]. Such interventions, which blend reactive agility, external focus, and multitasking, may facilitate the restoration of automatic motor control and improve long‐term injury resilience.

Although vertical jump assessments may be more sensitive for detecting persistent neuromuscular asymmetries after ACLR, the SLHD was selected because it is widely used in clinical RTS testing and combines lower‐limb propulsion, dynamic stability and landing control under functional single‐leg conditions [5, 56–58]. Furthermore, the SLHD was considered particularly suitable for integration within the present dual‐task paradigm. Future studies should compare horizontal and vertical jump tasks under dual‐task conditions to better characterise residual deficits after ACLR.

In summary, this study demonstrates that adding a cognitive task reveals subtle but clinically meaningful deficits in hop performance and movement quality that standard single‐task tests may overlook. Dual‐task testing may therefore serve as a valuable addition to late‐stage functional assessments, providing insight into the cognitive–motor readiness required for safe return to sport.

### 4.1. Limitations

The interpretation of these findings should be tempered by several methodological and practical constraints inherent to the study. The sample size, although comparable to prior dual‐task investigations in ACLR populations, remains modest and limits statistical precision, particularly for detecting small Group × Condition interactions, sequence/order effects or site‐related influences. Recruitment was constrained by multicentre coordination and strict eligibility criteria targeting individuals approximately 6 months after ACLR without concomitant lower‐limb pathology, which inevitably narrows external validity. Although linear mixed‐effects models were used to account for the repeated‐measures structure of the study and to adjust for sequence/order and centre effects, the limited sample size reduced the precision of model estimates, particularly for secondary outcomes and interaction terms. Future studies with larger samples should confirm these findings using prespecified mixed‐effects models and adequately powered primary outcomes.

The counterbalanced design allowed exploration of sequence‐specific responses and potential order, learning and fatigue effects. However, sequence‐specific findings were interpreted descriptively and should not be considered confirmatory. In particular, DTC was calculated only for the crossover sequences A1 and A2, in which both single‐task and dual‐task conditions were available within the same participant. Sequences B1 and B2 involved repeated assessments of the same condition and were therefore not interpreted as DTC sequences. This distinction should be considered when interpreting the magnitude and clinical meaning of the sequence‐specific findings.

Minor deviations from the registered protocol occurred during the analytical and reporting phases of the study. Specifically, condition‐specific analyses were reorganised to improve clarity between single‐task and dual‐task comparisons, and statistical outputs were rechecked following peer‐review comments, resulting in correction of several effect sizes and *p* values. In addition, one participant presented incomplete data for a specific sequence; available raw outcome data were retained using an all‐available‐data approach, whereas outcomes requiring both single‐task and dual‐task measurements were calculated using an available‐case approach. These modifications did not affect the experimental procedures or the overall interpretation of the study findings.

Outcome assessment relied on clinically pragmatic measures. SLHD is ecologically relevant but lacks the granularity of three‐dimensional motion analysis. Similarly, the QASLS, while supported by reliability literature and implemented with blinded video ratings, remains a semiquantitative visual scale that can be influenced by camera setup, rater calibration and subtle interpretive variability despite standardisation. The cognitive task combined with the motor trial primarily taxed working‐memory and verbal processes; other cognitive domains, such as visuospatial tracking, response inhibition or reactive decision‐making, might produce different interference patterns and cannot be inferred from the present protocol. The study captured a single postoperative time point, precluding inferences about the evolution of cognitive–motor interference across rehabilitation. It is plausible that dual‐task decrements attenuate with progressive exposure to complex environments or targeted neurocognitive training, but this requires longitudinal confirmation.

Rehabilitation exposure was not fully standardised across centres. Although all ACLR participants followed conventional postoperative rehabilitation, detailed information on training intensity, adherence, progression criteria and sport‐specific exposure was not available for all participants. This pragmatic variability may have influenced functional and dual‐task outcomes and should be considered when interpreting the findings. Contextual variability across sites, including flooring, ambient lighting, camera positioning and local assessor routines, may also have contributed unexplained heterogeneity in absolute values. Although condition order was counterbalanced and sequence allocation concealed, such environmental factors are difficult to eliminate in pragmatic settings and should be considered when generalising the results.

Taken together, these considerations argue for cautious interpretation of effect magnitudes while supporting the relevance of assessing cognitive–motor interference during late‐stage ACLR rehabilitation. The findings should be considered exploratory and hypothesis‐generating, particularly for secondary outcomes and sequence‐specific analyses.

## 5. Conclusion

Introducing a concurrent cognitive task during single‐leg hop testing revealed persistent limb symmetry alterations and clinically relevant cognitive–motor constraints in individuals approximately 6 months after ACLR. Although SLHD and QASLS DTCs were not significantly different between groups, limb symmetry appeared more affected under dual‐task demands in the ACLR group. These findings support the potential value of incorporating dual‐task assessments into late‐stage functional evaluations, while confirming that larger confirmatory studies are needed.

## Funding

No funding was received for this manuscript. Open access publishing was facilitated by Haute Ecole Specialisee de la Suisse Occidentale, as part of the Wiley‐Haute Ecole Specialisee de la Suisse Occidentale agreement via the Consortium Of Swiss Academic Libraries.

## Conflicts of Interest

The authors declare no conflicts of interest.

## Data Availability

The data that support the findings of this study are available upon request from the corresponding author. The data are not publicly available due to privacy or ethical restrictions.
